# Characterization of Laboratory Flow and Performance for Process Improvements via Application of Process Mining

**DOI:** 10.1055/a-1996-8479

**Published:** 2023-02-22

**Authors:** Eline R. Tsai, Andrei N. Tintu, Richard J. Boucherie, Yolanda B. de Rijke, Hans H.M. Schotman, Derya Demirtas

**Affiliations:** 1 Department of Clinical Chemistry, Erasmus University Medical Center, Rotterdam, The Netherlands; 2 Center for Healthcare Operations Improvement and Research (CHOIR), University of Twente, Enschede, The Netherlands; 3 Department of Clinical Chemistry, Amsterdam University Medical Center, VU Medical Center, Amsterdam, The Netherlands

**Keywords:** process mining, laboratory logistics, key performance indicators, clinical chemistry, process discovery

## Abstract

**Background** The rising level of laboratory automation provides an increasing number of logged events that can be used for the characterization of laboratory performance and process improvements. This abundance of data is often underutilized for improving laboratory efficiency.

**Objectives** The first aim of this descriptive study is to provide a structured approach for transforming raw laboratory data to data that is suitable for process mining. The second aim is to describe a process mining approach for mapping and characterizing the sample flow in a clinical chemistry laboratory to identify areas for improvement in the testing process.

**Methods** Data were extracted from instrument log files and the middleware between laboratory instruments and information technology infrastructure. Process mining was used for automated process discovery and analysis. Laboratory performance was quantified in terms of relevant key performance indicators (KPIs): turnaround time, timeliness, workload, work-in-process, and machine downtime.

**Results** The method was applied to two Dutch university hospital clinical chemistry laboratories. We identified areas where alternative routes might increase laboratory efficiency and observed the negative effects of machine downtime on laboratory performance. This encourages the laboratory to review sample routes in its analyzer lines, the routes of high priority samples during instrument downtime, as well as the preventive maintenance policy.

**Conclusion** This article provides the first application of process mining to event data from a medical diagnostic laboratory for automated process model discovery. Our study shows that process mining, with the use of relevant KPIs, provides valuable insights for laboratories that motivates the disclosure and increased utilization of laboratory event data, which in turn drive the analytical staff to intervene in the process to achieve the set performance goals. Our approach is vendor independent and widely applicable for all medical diagnostic laboratories.

## Background and Significance

The rising level of laboratory automation provides an increasing number of logged events, yielding accurate timestamps and valuable data of the automated laboratory processes. These data are important for characterizing laboratory performance and process improvements. Unfortunately, this abundance of data is underutilized, 1 2 which might be due to laboratory staff being unaware how to use this data to characterize laboratory processes, or data not being available in an easily accessible or interpretable format.

Process mining enables automated discovery and visualization of process-related information and process models based on event logs without a priori information about the process, with the aim to discover, monitor, and improve these processes. 3 This is in contrast with manual constructions of process models, such as, for example, done in Yu et al. 4 Sample flow can be complex, making the use of process mining to reveal the paths of the samples in the testing process a promising strategy. This allows laboratory technicians to have a better understanding of the paths followed by the samples. A recent review reports 270 articles in which process mining is applied in a health care context. 5 The two most reported contributions to health care are related to process discovery and analysis for the evaluation of patient care and assessing the compliance to protocols and guidelines. 5 Cancer and emergency care are found to be the two most common application areas in health care. 6 Other example applications are mapping the diagnostic paths for patients with nonspecific abdominal pain 7 and mining the flow of pediatric trauma patients. 8 There are few studies which reported process mining in conjunction with medical diagnostic laboratories. These studies either report the laboratory as a single activity in the process map, 9 10 11 12 13 or only report the type of blood tests performed. 14 15 Despite the increasing popularity of using process mining in a health care setting, there are no studies on applying process mining within a medical diagnostic laboratory, including activities in the different subprocesses within the laboratory.

Three types of process mining may be distinguished 3 : (1) discovery, in which a process model is constructed solely based on the information in an event log; (2) conformance, where it is checked whether the process according to the event data conforms to an existing process model; and (3) enhancement, in which an existing process model is extended or improved based on event data. In this study, we use process discovery and enhancement.

It is crucial to identify relevant key performance indicators (KPIs) to characterize the performance of the diagnostic process and identify areas for improving laboratory efficiency. 16 In this study, we also use process mining to characterize the process via relevant KPIs. Turnaround time (TAT) is the most frequently reported KPI to quantify laboratory performance. 16 Timeliness of results is an important laboratory KPI as it can affect the quality of care through timely physician action, 16 such as providing swift medical action in case of critical results. 17 Other relevant KPIs considered in this article are work-in-process, workload, and machine downtime. 16

### Objectives

The aims of this descriptive study are to provide a structured approach for transforming raw laboratory data to data that is suitable for process mining, and to describe a process mining approach for mapping and characterizing the sample flow in a clinical chemistry laboratory to identify areas for improvement in the testing process.

## Methods

### Study Setting

**Fig. 1:**
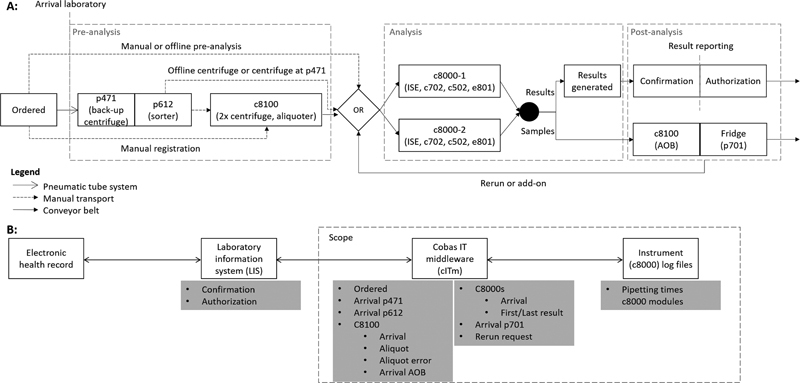
**(A)** Schematic overview of main sample flow at Erasmus MC. **(B)** Connection of the activities to the information technology (IT) infrastructure.

The testing process of a Dutch clinical chemistry laboratory is considered, and is detailed for Erasmus MC, University Medical Center Rotterdam (Erasmus MC). A schematic overview of the main sample flow ( Fig. 1A ) and the connection of the activities to the information technology (IT) infrastructure ( Fig. 1B ) are shown. Preanalysis consists of the p471, p612, and cobas 8100 (c8100). The p471 is the back-up centrifuge and the p612 is the sorter. The c8100 contains the two main centrifuges. It also contains an aliquoter, which generates secondary sample tubes, called aliquots, from the collection tubes. Aliquots are used to reduce the risk of carry-over, or are made when a collection tube requires testing on multiple analyzer lines. Analysis consists of two cobas 8000s (c8000) analyzer lines which in turn consist of four modules, ISE, c702, c502, and e801, on which the actual diagnostic testing is performed. Erasmus MC has a centralized sample reception facility for all laboratories in the hospital. Non-c8000 samples are meant for other laboratories and are only processed on the preanalytical instruments, and hence are out of scope for this study. Supplementary Appendix A (available in the online version)provides a more detailed description of the testing process. Erasmus MC uses Roche Diagnostics instruments (Roche Diagnostics International Ltd, Rotkreuz, Switzerland).

### Data Collection

Data were obtained from the cobas IT middleware (cITm, Roche Diagnostics International Ltd) and the c8000 log files. The c8000 log files contain data directly exported from the data manager of the c8000's and cITm is the middleware between laboratory instruments and laboratory information system (LIS). The c8000 log files contain per sample and per test the pipetting times and the cITm data contain per sample and per test the date and time of various activities ( Fig. 1B ). The collected timestamps are on a precision level of seconds. We collected data from January 2019, which is one of the busier months in the laboratory, allowing sufficient accuracy of characterization of the testing process. The data contained information on all samples processed on the instruments in Fig. 1A during this time period.

### Data Preparation

**Fig. 2:**
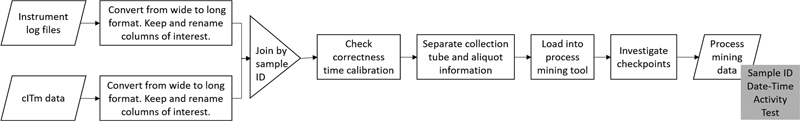
Data preparation process.

The data preparation process is visualized in Fig. 2 . Reformatting, joining by sample ID, checking correctness time calibration, and separating aliquot and collection tube information were performed in R 3.4.4 18 via RStudio Desktop. 19 To combine cITm and c8000 log files reformatting was required as each have their own data format. The clocks in cITm and the c8000 data manager are not always properly aligned and therefore timestamps needed to be corrected before data analysis. This step can also be performed before joining the data sets, or incorrect time calibration can be checked when the testing process is visualized via process mining. Collection tube and aliquot information were separated and assigned different IDs as these are two separate flows from aliquoting onward.

Except for a few manual steps, such as potential manual registration and manual transport between the two preanalysis modules p612 and c8100, the testing process is fully automated. Therefore, all samples should include the sample-ordered timestamp, the arrival of the sample at an analyzer line, and the generation of test results ( Table 1 ). We check how many samples contain these timestamps, referred to hereinafter as the checkpoints, in their event log.

**Table 1:** Description of the three checkpoints, which should occur in the event logs of all the samples

Checkpoint	Description
Ordered	The time that the sample order is known in cITm, a which occurs after the tests are ordered by the physician. The first “ordered” timestamp occurs before the first scan moment in the laboratory, but for add-ons the “ordered” timestamp can occur later in the testing process, depending on when these additional tests are ordered
C8000-x	The time that the sample enters an analyzer line for testing. The “x” is either 1 or 2, and stands for one of the two analyzer lines
Result	The time that the results are generated

a Cobas information technology (IT) middleware.

Additional data preparation is required to analyze subgroups, such as the subgroup of samples of which the results are not delivered on time. For the KPI timeliness, we used arrival to p471 as the start point, since this is the first logged event in the laboratory, and the time that the results are generated as the final point, as it is the last logged event in the data that affects the TAT. Sometimes, additional tests are ordered while a sample is already in the laboratory for initially ordered tests. In this case, we exclude such samples from the timeliness analysis since it is not possible to filter out the results of the added tests, which may be added hours or even days later. Therefore, the time-related KPIs TAT and timeliness are only applicable to the initial round of tests. Furthermore, as collection tube and aliquot information was split and aliquot event logs do not contain the p471 timestamp, timeliness was only checked for the collection tube.

### Process Discovery, Enhancement, and Analysis

We used the process mining tool Disco whose miner is based on the Fuzzy miner and is further developed based on user experience. 20 The Fuzzy miner is used for automated discovery of the different paths of the samples in the testing process based on historical data. Then, the process mining tool is used to extract relevant KPIs. For example, the process map is enhanced with frequency and duration information to get insights into the KPIs TAT and workload. This type of enhancement is called “extension.” 3 Process mining tools also have built-in filtering options and can thus be used to further inspect and clean the data. A brief description of the Fuzzy miner and alternative process mining tools and functionalities are provided in online Supplementary Appendix B ( Supplementary Table S1 , available in the online version) and and Supplementary Appendix C ( Supplementary Figs. S2 S3 , available in the online version).

Table 2 describes five KPIs used to characterize the testing processes, including how these were analyzed. These KPIs are crucial for characterizing and monitoring the performance of the testing process.

**Table 2:** Description of the five KPIs taken from Tsai et al and Steindel and Howanitz ^16,27^ including how these were analyzed

KPI	Description	How analyzed
Machine downtime	Downtime occurs when a machine is not working, which can be due to machine failure or scheduled maintenance	Plotting the number of events over time and observing periods with no activity
TAT	The time between the moment that the sample arrives at the laboratory until the results are reported, which is the most commonly used definition of TAT	Enhancing the process map with duration information to analyze the time between subsequent steps as these affect the TAT
Timeliness	A test result is timely if its TAT is less than the predefined TAT target	Filtering the data such that samples not meeting the TAT target are shown
Work-in-process	The work-in-process is the number of samples in the process, at a given time, that have not yet finished testing	Plotting the active cases over time
Workload	The workload is the number of samples, or tasks, assigned to a laboratory resource over a certain period	Plotting the number of events over time and enhancing process map with case frequency duration. Case frequency corresponds to how many samples performed an activity or traversed an edge

Abbreviations: KPIs, key performance indicators; TAT, turnaround time.

## Results

**Fig. 3:**
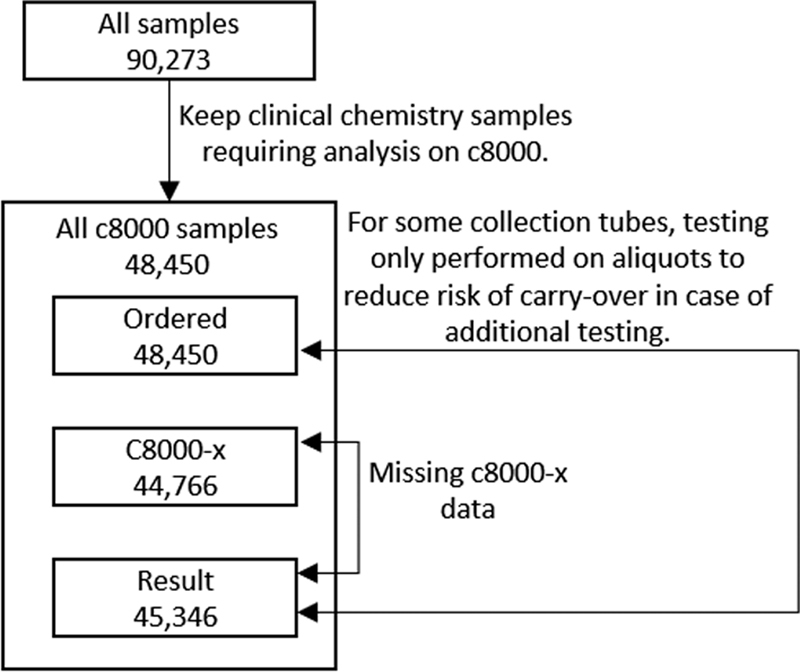
Number of samples containing the checkpoints, which should occur in the event log of all the samples.

**Fig. 4:**
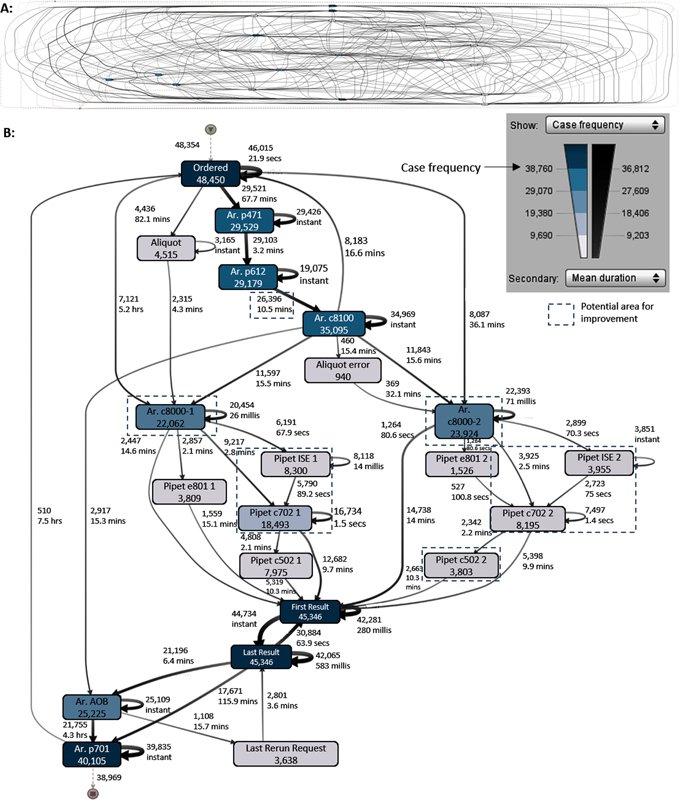
**(A)** Process map showing all paths. **(B)** Process map showing the top 10% most dominant paths. Based on January 2019 data, including case frequency and mean duration. For events with “Ar.” (arrival) the outgoing arrows include the processing time of this event, else the entering arrows contain the processing time.

We applied process mining to the Erasmus MC data of January 2019 and analyzed the KPIs of interest. Checkpoints for analysis of Erasmus MC laboratory are shown in Fig. 3 . The data contained 90,273 samples, including non-c8000 samples. After removing the non-c8000 samples, 48,450 samples remained, of which 92% contained all the checkpoints. We still included the samples with the missing checkpoints as the other activities in their event logs were still representative. Fig. 4A shows the process map containing all the paths taken by these samples, demonstrating the complexity of the testing process. To enhance readability and interpretability, Fig. 4B shows the process map for the top 10% most dominant paths including case frequency and mean duration. Supplementary Fig. S1 in Supplementary Appendix A (available in the online version) indicates the proximity of the equipment. From the process map, we observe that samples have taken many different routes along the laboratory instruments. The ISE, c702, and e801 modules were mostly visited before the c502 module. We can also identify paths deviating from the main sample flow, such as the path going from c8100 to “ordered” which corresponds to serum indices being added to the order of a sample while the sample is in the c8100.

The incubation process at a module is done in parallel with the pipetting and incubation processes at subsequent modules on a rack's route. Approximately 40% of the samples are not routed from highest to lowest incubation time, even though they could. The process map is enhanced with duration information between subsequent events. The times between the events include waiting, processing, transport, and scanning times of the samples. Fig. 4B shows that the mean duration between arrival at p612 and arrival at c8100 was 10.5 minutes, where these machines are just 2 m apart and the transport is done manually. The process map is also enhanced with frequency information, from which we observe that more samples were tested on the c8000-2 (23,924 samples, 196,601 tests) as compared to the c8000-1 (22,062 samples, 175,158 tests). It is noteworthy that the setup of the two analyzer lines and their installed reagents is almost identical, with reagents of seldomly ordered tests only installed on one analyzer line.

**Fig. 5:**
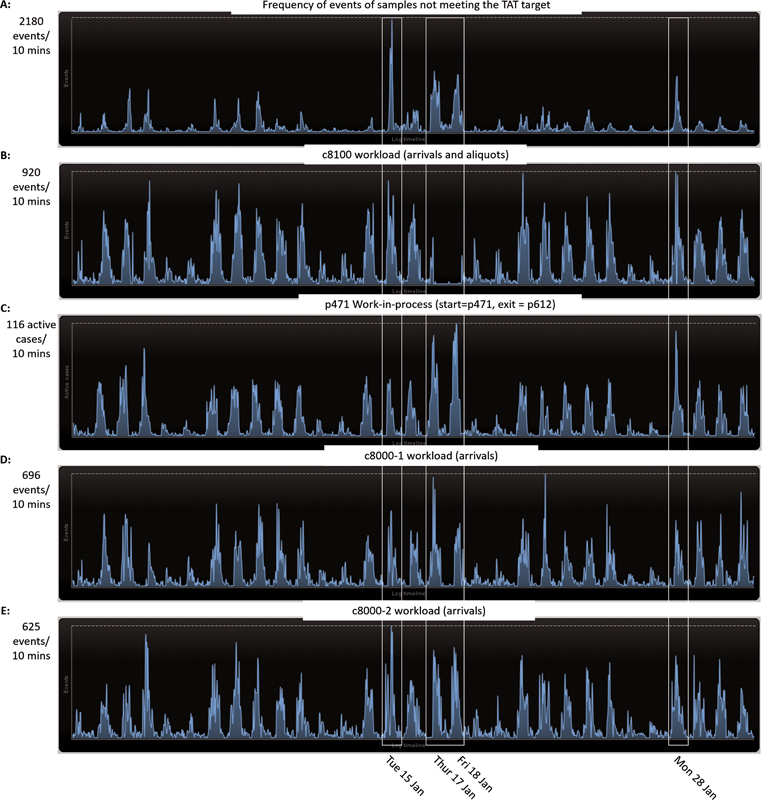
**(A)** Frequency of events of samples passing the through p471 whose results generation was not timely. **(B, C)** Relation between workload of c8100 and work-in-process of p471 of all samples, showing downtime of c8100 on January 17, 18, and 28. **(D, E)** Workload c8000-1 versus workload c8000-2 of all samples, showing downtime on both c8000s on January 15. Data from January 2019.

Erasmus MC has a TAT target of 60 minutes for both high priority and routine samples. We further analyzed the samples whose results generation was not timely. Results of 12.5% of the regular samples and 5% of the high-priority samples were not generated on time. In particular, more samples did not meet the TAT target on days on which there was machine downtime. For instance, on January 15, 17, 18, and 28 there were peaks in the number of samples not meeting the TAT target ( Fig. 5A ). Due to machine failure, the c8100 was mostly offline on January 17 and 18 ( Fig. 5B ). This resulted in a peak in the work-in-process on the back-up centrifuge on the p471 ( Fig. 5C ). During this period, results of 52 of 235 high-priority samples and 948 of 1,951 regular samples were not generated on time. These samples make up 22% of the high-priority and 34% of the regular samples whose results generation was not timely in general. Furthermore, the c8000s were partly offline on January 15, which can be observed by the sharp decrease around noon where the workload reaches 0 events/min ( Fig. 5D and E ). Finally, on January 28 there was downtime of the c8100 at midday, which again can be observed by the sharp decrease around noon ( Fig. 5B ).

Application to a second clinical chemistry laboratory, namely the Amsterdam University Medical Center, location VU Medical Center (VUmc), is included in online Supplementary Appendix D ( Supplementary Figs. S4 – S7 , available in the online version).

## Discussion

Process mining has successfully been applied in various health care settings. 5 6 21 Here, we provide the first application of process mining to the data within a medical diagnostic laboratory for automated process model discovery, showing its benefits and managerial impact. The testing processes in two clinical chemistry laboratories were characterized and bottlenecks and areas for improvement were identified based on relevant KPIs. The developed approach is vendor independent and widely applicable for medical diagnostic laboratories, and potentially beyond, to improve the efficiency of the testing process. Ultimately, the insights from this research are used to set up studies to optimize the laboratory logistics.

We provide three main takeaways based on the laboratory processes from this descriptive study. (1) Process mining can be used to analyze the paths followed by the samples to identify areas where alternative routes might increase laboratory efficiency. We have shown in our previous study that routing from high to low incubation time provides the optimal TAT for laboratories, 22 as the incubation process at the previous module and pipetting at subsequent modules is done in parallel. However, our process mining analysis showed that 40% of the samples do not obey this rule, even though they could. To reduce the TAT, the routes should be redesigned considering incubation times. Our process mining approach can then be used again to compare performance before and after the interventions. (2) Process mining enables laboratories to review preventive maintenance policies and backup processes during machine downtime. When the percentage of (high-priority) samples whose results generation was not timely during machine downtime is higher than a predefined target, the laboratory should take action. Our analysis revealed that during malfunction of the c8100, results of 52 of 235 high-priority samples and 948 of 1,951 regular samples were not generated on time even though there is a backup centrifuge. These samples make up 22% of the high-priority and 34% of the regular samples whose results generation was not timely in general. Therefore, the laboratory should review the preventive maintenance policy to increase reliability of the laboratory instruments. Furthermore, since the results of a large number of high-priority samples were not generated on time during machine failure, the laboratory should review the routes of these samples during the backup process to decrease their TAT during downtime. (3) By visualizing the testing process and enhancing the process map with KPIs, irregularities in the testing process can be observed which trigger further research to improve efficiency of the diagnostic process. For example, the time spent between arrival at p612 and c8100 is unexpectedly high, amounting to 10.5 minutes, which may be due to manual transport of the samples over the 2-m distance between the p612 and c8100. Reducing waiting time for manual transport is a feasible and straightforward measure to reduce TAT. We also found that the workload of the analyzer lines was unbalanced. The setup of the two analyzer lines and their installed reagents are almost identical. Therefore, one might expect a more balanced workload to reduce TAT. More research is needed to investigate reasons and impact of these irregularities. In ongoing research, we use mathematical modeling to optimally assign samples to the analyzer lines to reduce TAT.

The Fuzzy miner used in this research is very suitable to handle large event logs, highly unstructured processes, and allows for different simplified views of the process, which considerably contributes to interpretability of the resulting process map. 20 The Heuristic miner 23 and the Fuzzy miner 24 are reported as the two most commonly used miners for process discovery in health care. 5 6 21 A limitation of our study is that the Fuzzy miner is less favorable for processes in which parallel processing occurs. Due to this limitation, we split collection tube and aliquot information and measure the TAT only for the collection tube, which underestimates TAT. Another limitation of our study is that for most parts of the testing process, the data only contain the arrival timestamp, which prevents us from differentiating between the transport, setup, waiting, and processing times inside the instruments. Therefore, the analysis for the time spent in the c8100 is limited. Logging more subprocesses in the laboratory would allow one to determine how much time is allocated to the different parts of the processes, and consequently to obtain additional insights into bottlenecks and inefficiencies within the instruments. We analyzed 1 month of data as a proof of concept, which is another limitation as this does not allow analysis of seasonal patterns. The developed approach can easily be extended to analyze seasonal patterns.

Process mining solutions that offer continuous monitoring of the processes under consideration are an emerging trend. 25 26 An interesting extension of our work is the real-time monitoring of the paths of the samples, which is appealing for taking timely actions to achieve the TAT goal. Other interesting extensions are to use our approach for analyzing the effects of laboratory errors, different levels of aggregation and different subpopulations, and the technical confirmation and medical authorization process. For example, applying our approach to samples containing a particular laboratory error can help to determine how and to what extent these errors affect laboratory performance. Moreover, one can obtain insights into the test ordering behavior by reformatting the input data such that the activities represent the performed tests instead of the instruments visited, such as in Suriadi et al. 15 Furthermore, data from the LIS, which contain information on the technical confirmation and medical authorization process, was excluded due to missing information for aliquots and reruns in the LIS. In future research, once these issues are resolved, by including the technical confirmation and medical authorization process, one could obtain a more comprehensive understanding of the testing process.

## Conclusion

Sample flow in medical diagnostic laboratories is complex. As a consequence, laboratory technicians may lack a clear idea about the paths followed by the samples, making laboratory optimization challenging. Process mining can empower laboratories to visualize their testing process based on historical data in an automated manner. Combined with the use of relevant KPIs, it allows laboratories to characterize and analyze their testing process to identify bottlenecks and areas for improvement, which paves the way to increase the efficiency of the diagnostic process.

## Clinical Relevance Statement

The developed process mining approach helps laboratories to understand the complex sample flow in the diagnostic process. Combined with the use of relevant KPIs, the performance of the testing process is characterized and areas for improving laboratory efficiency are identified.

## Multiple-Choice Questions

1. Which of the following types of process mining can be described as “extending the process map with information from the event log about the process under consideration, such as including frequency and duration information”?
  - Discovery
  - Enhancement
  - Conformance
  - None of the above
  **Correct Answer:** The correct answer is option b. Enhancement is the type of process mining in which an existing process model is extended or improved based on event data.
2. What type of information is at least needed for such studies, i.e., to mine the logistics in the diagnostic process?
  - Ordered test + timestamp + event
  - Sample ID + priority + event
  - Sample ID + ordered test + timestamp
  - Sample ID + timestamp + event
  **Correct Answer:** The correct answer is option d. To analyze the paths followed by the samples, one requires to know which sample (sample ID) was doing what (event) at what time (timestamp). Including the priority and ordered test provide useful insights but are not required for the purposes of this study.
