# Supplementary material for: Characterization of Laboratory Flow and Performance for Process Improvements via Application of Process Mining
Source: Appl Clin Inform. 2023 Feb 22;14(1):144–52. doi: 10.1055/a-1996-8479 (PMC9946784; doi:10.1055/a-1996-8479)
Supplement: Supplementary file 1 — Supplementary Material [file 10-1055-a-1996-8479-s202206ra0168.pdf]

Supplementary Appendix A: Detailed Study Setting Description

In this section we elaborate on the testing processes of Erasmus MC (►Fig. 1A) and VUMC (►Supplementary Appendix D). Both laboratories use Roche Diagnostics instruments. As an example, the layout of the clinical chemistry laboratory in Erasmus MC is shown in ►Supplementary Fig. S1.

Samples arrive in the laboratory via the pneumatic tube system or per courier. The samples are then loaded into the p471 where their arrival is automatically registered. Additionally, samples can also be manually registered by a laboratory technician. In case of manual registration, samples can skip the p471 and p612 prior to manual loading into the c8100.

The preanalytical phase comprises of two parts:

1. The first part consists of the p471 and the p612. The p471 contains a centrifuge, which is used for backup or for the centrifugation of samples that have to rest for a while before they are ready for analysis. In the p612, samples are sorted based on their priority level and destination laboratory. In contrast to VUmc, the p471 in Erasmus MC functions as a centralized sample reception facility for all laboratories in the hospital and therefore does not only process clinical chemistry samples. After the first part, samples are manually transported to the second part of the preanalytical phase.
2. The second phase consists of the c8100 in Erasmus MC and MPA in VUmc. The c8100 and MPA consist of two centrifuges, a destopper, an aliquoter, and a labeller. In the MPA samples are routed in 5-position racks, while in the c8100 they are transported in single tube holders.

The collection tube and its aliquots are routed separately through the testing process. The results from both collection tube and its aliquots should be available before the results enter the postanalytical phase.

Both laboratories have two c8000 analyzer lines for sample analysis, which are connected to the second part of the preanalytical phase via a conveyor belt. In the c8000s samples are routed in 5-position racks. In Erasmus MC, samples are converted from single tube holders to 5-position racks before entering a c8000 line, which is done in the BRF. The analyzer lines contain one ion-selective electrode (ISE) module, two chemistry modules (c702 and c502), and one immunochemistry module (e801). The test-mix of a sample determines which analyzer modules a sample must visit.

In Erasmus MC, when samples have visited all the required modules, they are routed back to the c8100 to be temporarily stored in the add-on/output buffer module (AOB) to allow for quick access when reruns or add-ons are requested. After 3 hours they are transported via a conveyor belt to the p701 cold storage where they are stored for 5 days before being automatically disposed. In VUmc, samples are manually transported from the c8000 to an offline fridge, where they are stored for 3 days before being disposed. Samples are stored in case add-ons or reruns are requested.

In the meantime, the generated test results go through confirmation (technical validation), authorization (medical validation), and result reporting.

Supplementary Appendix B: Fuzzy Miner and Alternative Process Mining Tools

In this section we give a brief description of the Fuzzy miner and alternative tools that can be used for process discovery and analysis.

The Fuzzy miner uses significance and correlation metrics to develop decision criteria to use in the simplification and visualization of process models.[1] To simplify the process model, highly significant behavior is preserved, less significant but highly correlated behavior is aggregated, and less significant and lowly correlated behavior is removed from the model. The desired cutoff values can be specified by the user.

The approach described in this article can also be performed with other process mining tools. The Fuzzy miner is also, for example, built into the process mining tool ProM. We used Disco as it allows the user to easily adjust the percentage of activities and paths shown in the visualization. The best setting of these filters depends on the use of the process map. ►Supplementary Table S1 points out some of the differences between ProM and Disco.

In the main text we reported mean durations. Process mining tools allow us to obtain more duration statistics, on which we elaborate in ►Supplementary Appendix C.

References

1. Günther CW, Van Der Aalst WMP. Fuzzy mining - adaptive process simplification based on multi-perspective metrics. In: International Conference on Business Process Management; 2007:328-343
2. Leemans S, Fahland D, Aalst WMP van der. Process and Deviation Exploration with Inductive Visual Miner. BPM (demos) 2014; 1295(8)

Supplementary Appendix C: Detailed Duration Statistics

Process mining tools allow us to obtain more duration statistics than just the mean. This section elaborates on additional duration information obtainable by process mining tools. As an example, we focus on the time between arrival at p471 and p612 in Erasmus MC. The mean duration between these activities is 3.2 minutes (main text ►Fig. 4B), with a median of 71 seconds, minimum of 23 seconds, and maximum of 113.2 minutes. The distribution of the time between these activities is shown in ►Supplementary Fig. S2. ►Supplementary Fig. S2B zooms in on the samples with a duration of 5 minutes or longer between arrival at p471 and p612. As centrifugation takes 5 minutes, we know that samples with a duration less than 5 minutes between arrival at p471 and p612 are not centrifuged at the p471.

Duration information of individual samples can be obtained and exported to create boxplots. ►Supplementary Fig. S3

 © 2023. The Author(s).

shows that the median duration of all samples (1.18 minutes, ►Supplementary Fig. S3A) versus the samples not meeting the TAT target (1.87 minutes, ►Supplementary Fig. S3A) lie close to each other, but relatively more samples have a duration of more than 5 minutes in the latter case (►Supplementary Fig. S3B and S3C).

Supplementary Appendix D: Case Study: VUMC

We applied our method to the clinical chemistry laboratory of Amsterdam University Medical Center, location VU University Medical Center, Amsterdam (VUmc). A schematic overview is shown of the main sample flow (►Supplementary Fig. S4A) and connection of the activities to the IT infrastructure (►Supplementary Fig. S4B).

Data were collected from September 2020 and prepared according to Sections 2.2 and 2.3. Some adjustments are to be noted. At VUmc, log files start with a "sample added" timestamp, which corresponds to the registered arrival time of a sample in the laboratory. This is used as the start point for the KPIs TAT and timeliness. Similar to Erasmus MC, the time that the results are generated is used as the final point as it is the last logged event in the data that affects the TAT. Furthermore, contrary to Erasmus MC, p471 and p612 were not available.

Checkpoints for analysis are shown in ►Supplementary Fig. S5. The data contained 16,669 samples, including non-c8000 samples. After removing the non-c8000 samples, 16,276 samples remained, of which the vast majority (99%) contained all the checkpoints. We included all 16,276 samples in our analysis. ►Supplementary Fig. S6A shows the process map containing all the paths taken by these samples and ►Supplementary Fig. S6B shows the process map for the top 20% most dominant paths including case frequency and mean duration. From this process map, we can identify paths deviating from the main sample flow, such as the path starting at "aliquot" and continuing to "sample added." This is because in VUmc aliquots receive the "sample added" timestamp after they are made. We also observe that there are frequently traversed edges from "Result" back to the c8000s, since at VUmc the "c8000" is logged both when a sample enters and exits an analyzer line. The ISE, c702, and e801 modules were mostly visited before the c502 module. Similar to Erasmus MC, we observe that 42% of the samples did not visit the analyzer modules in decreasing order of the incubation times, which potentially increases TAT.[1] We also observe an unbalanced load for c8000-1 (9,186 samples) and c8000-2 (7,755 samples). More tests were installed on the e801 module of the c8000-1 as compared to the c8000-2, which is a tradeoff between costs and TAT. Further research is required to investigate whether assigning more samples, which can be tested on both analyzer lines, to c8000-2 results in a decrease in TAT.

VUmc has a TAT target of 60 minutes for both high-priority and routine samples. We further analyzed the samples whose results generation was not timely. Results of 456 of 7,221 regular samples and 241 of 5,268 priority samples were not generated on time. ►Supplementary Fig. S7A shows the frequency of events of the samples not meeting the TAT target. To investigate potential causes for samples not meeting the TAT target, workload and work-in-process plots were considered. On September 21 a relatively high number of samples arrived (►Supplementary Fig. S7B). As a result, we observe a slightly higher work-in-process prior to the MPA (►Supplementary Fig. S7C) and samples not meeting the TAT target (►Supplementary Fig. S7A). On September 28 there was downtime on the c8000-1 (►Supplementary Fig. S7D) and on September 9 there was downtime on the c8000-2 (►Supplementary Fig. S7E), resulting in an increase in the workload on the other analyzer line and delayed samples.

References

1. Tsai ER, Demirtas D, Tintu AN, de Jonge R, De Rijke YB, Boucherie RJ. Optimal design of networks of FIFO queues and Incubation queues with an application to the design of clinical chemistry laboratories (unpublished results)

 © 2023. The Author(s).

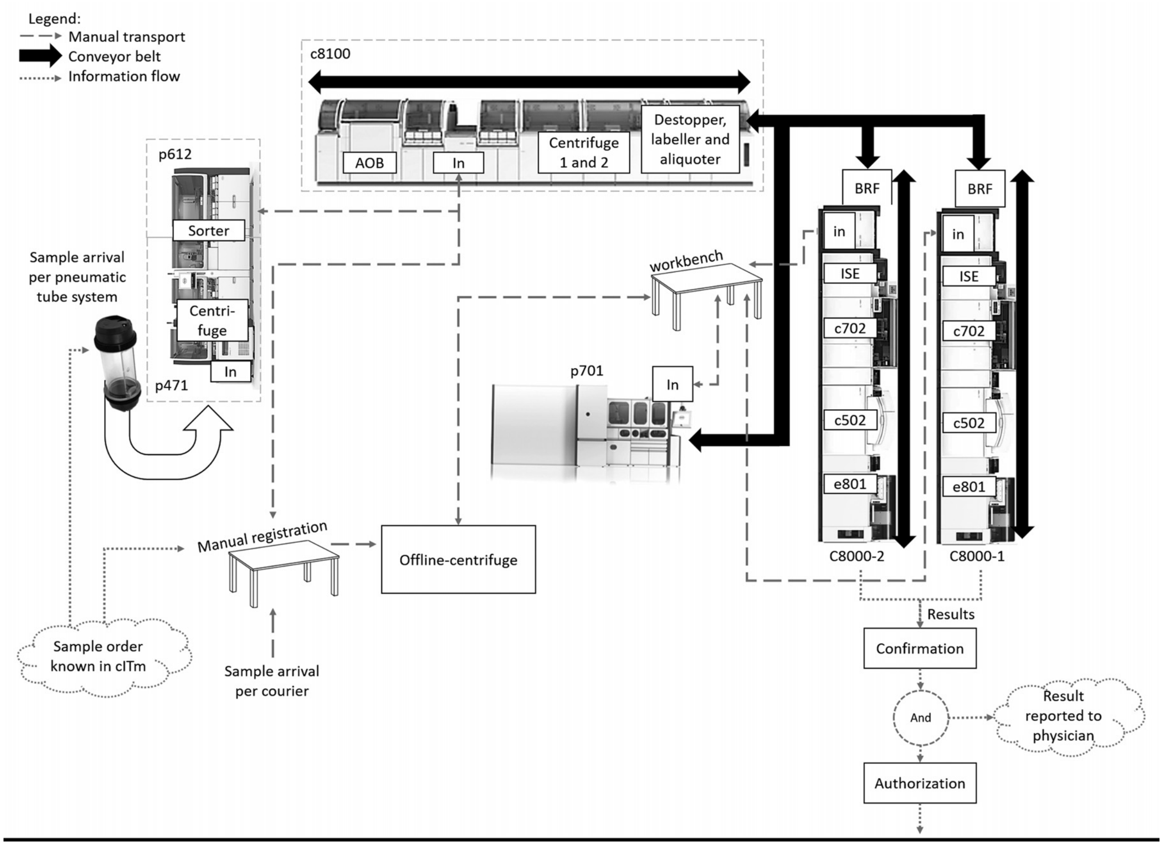

Supplementary Fig. S1 Layout of Erasmus MC clinical chemistry laboratory.

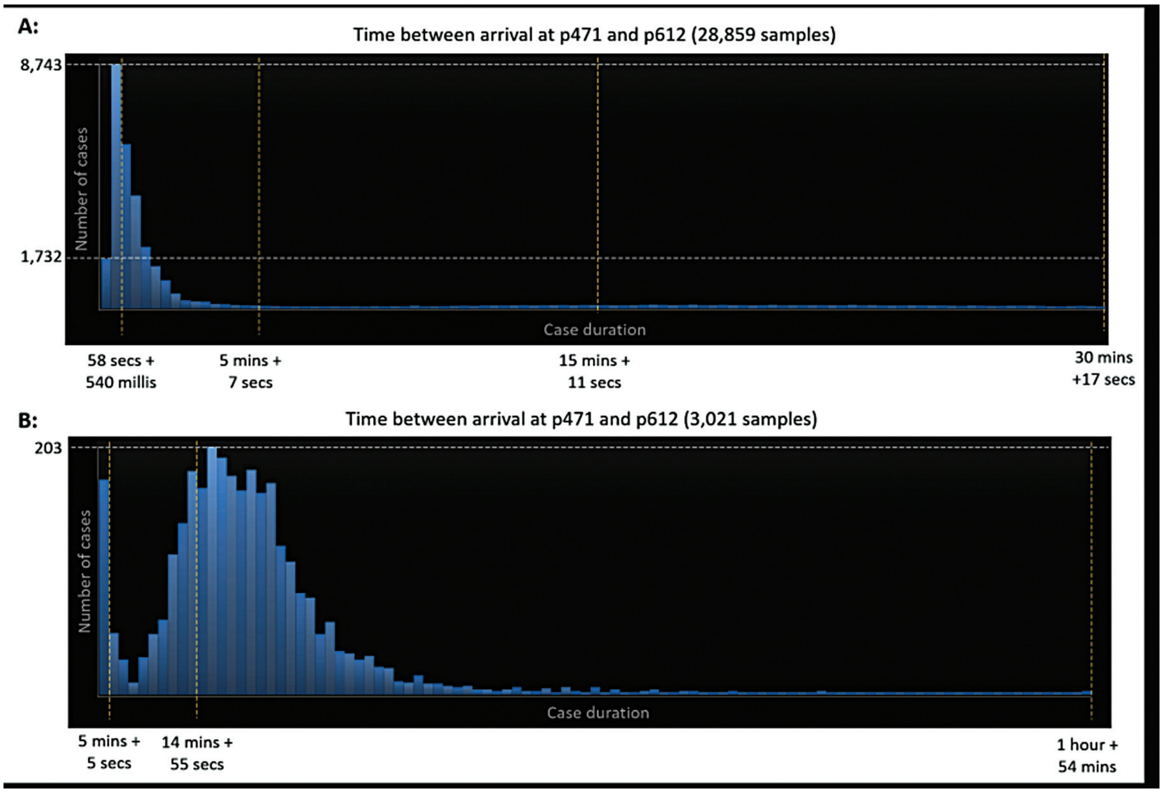

Supplementary Fig. S2 (**A**) Distribution of the duration between arrival at the p471 and arrival at p612 in Erasmus MC, including samples with a duration of at most 30 minutes. (**B**) Distribution of the duration between arrival at the p471 and arrival at p612 in Erasmus MC, including samples with a duration of more than 4 minutes.

 © 2023. The Author(s).

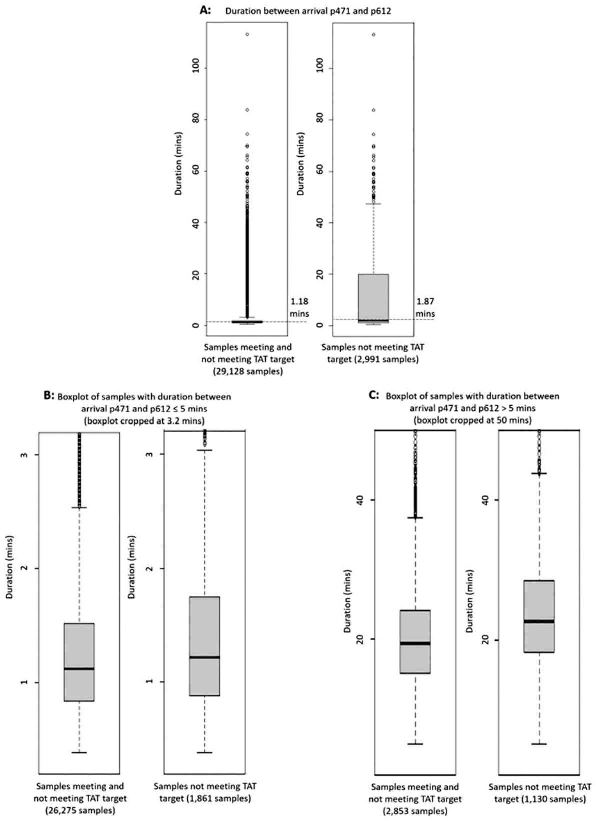

Supplementary Fig. S3 (**A**) Boxplot of the duration between arrival at the p471 and p612 in Erasmus MC based on January 2019 data. (**B**) Boxplot including samples with a duration of at most 5 minutes between arrival at p471 and p612. (**C**) Boxplot including samples with a duration of more than 5 minutes between arrival at p471 and p612.

 © 2023. The Author(s).

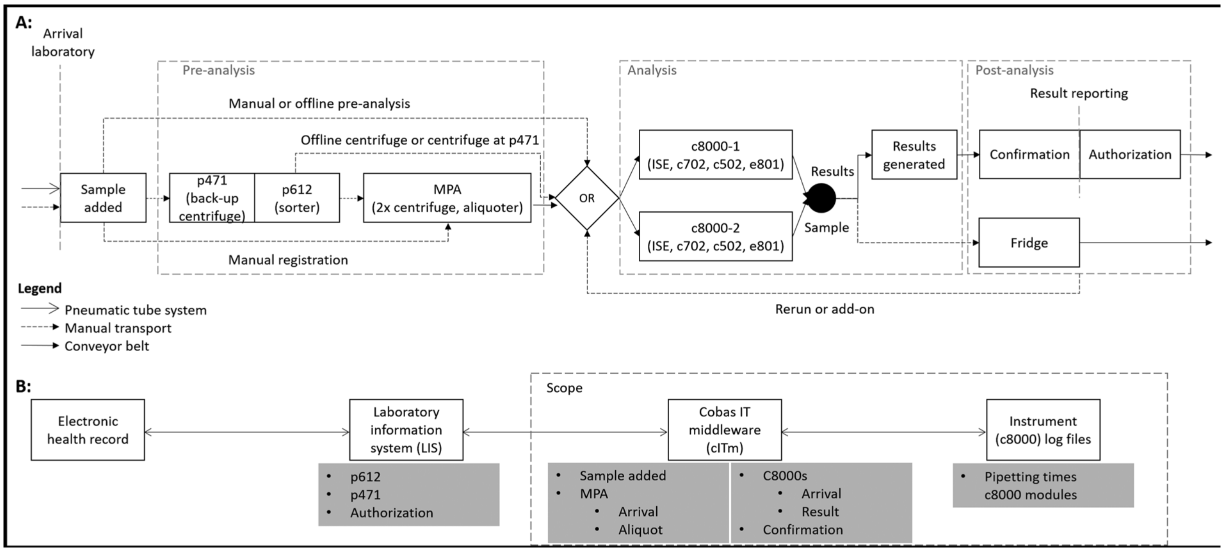

Supplementary Fig. S4 (A) Schematic overview of main sample flow in VUmc. (B) Connection of the activities to the information technology (IT) infrastructure.

 © 2023. The Author(s).

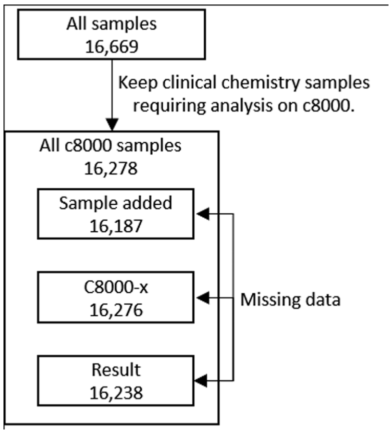

Supplementary Fig. S5 Number of samples containing the checkpoints, which should occur in the event log of all the samples.

 © 2023. The Author(s).

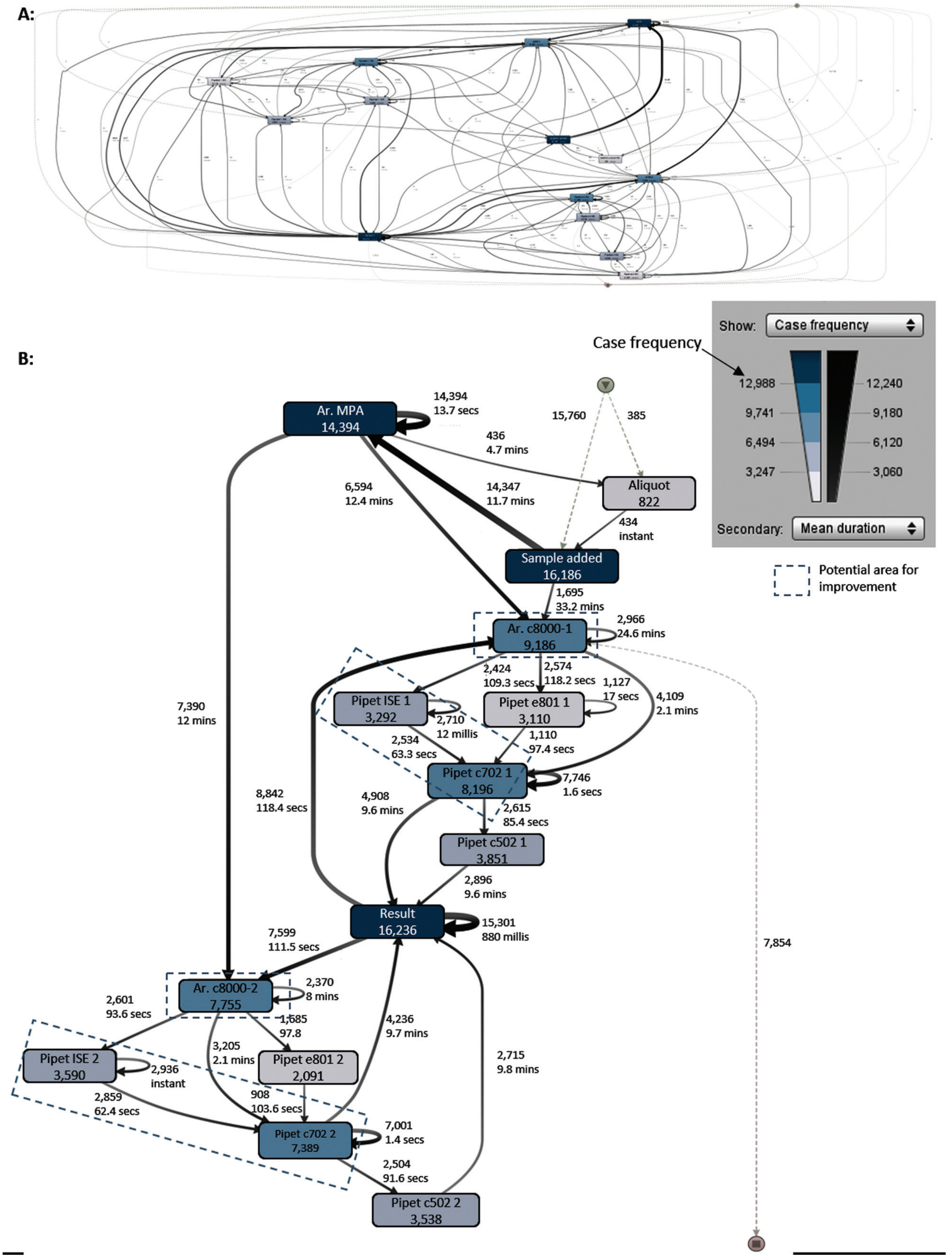

Supplementary Fig. S6 (**A**) Process map showing all paths. (**B**) Process map for VUmc showing the top 20% most dominant paths. Based on September 2020 data, including case frequency and mean duration. For events with "Ar." (arrival) the outgoing arrows include the processing time of this event, else the entering arrows contain the processing time.

 © 2023. The Author(s).

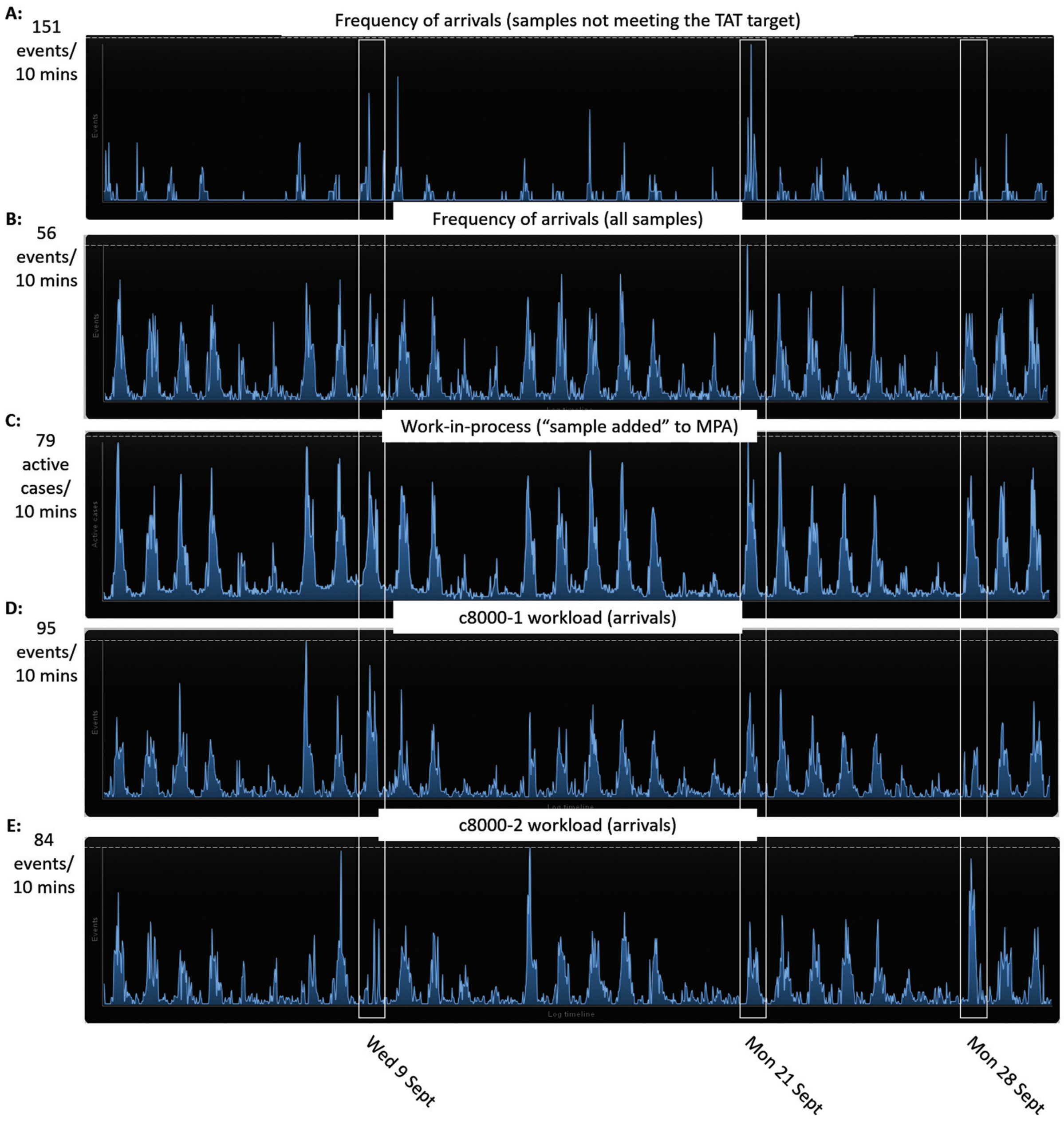

Supplementary Fig. S7 (**A**) Frequency of events of samples whose results generation was not timely. (**B**) Frequency of arrivals, including all samples, showing a high number of arrivals on September 21. (**C**) Work-in-process from “sample added” to MPA, showing a slightly higher work-in-process on September 21. (**D, E**) Workload c8000-1 versus workload c8000-2 of all samples, showing downtime of c8000-1 on September 28 and downtime of c8000-2 on September 9. Data from September 2020.

 © 2023. The Author(s).

Supplementary Table S1 Comparison of Disco and ProM

	Disco	ProM
Contains the Fuzzy miner?	Yes	Yes
Contains miners other than Fuzzy miner?	No	Yes
Additional data filtering possible?	Yes, in the "Filter" section	For example, using the plug-ins "filter events" by S. Leemans and "filter log by attributes" by D. Fahland
Possible to extend process map generated using Fuzzy miner with frequency and duration information?	Yes	No. However, it is possible when using the Inductive visual Miner by S. Leemans[2] or a miner that produces a process graph that can be converted into a petri net
Level of detail that can be adjusted.	Percentage of activities and paths shown	Various additional settings can be customized such as the weight put on endpoint correlation, which measures the correlation of two activities by the similarity of their labels
Uses aggregation as a form of process map simplification?	No	Yes

 © 2023. The Author(s).
